# Case Study of Diesters of o-Phthalic Acid in Surface Waters with Background Levels of Pollution

**DOI:** 10.3390/toxics11100869

**Published:** 2023-10-19

**Authors:** Alexander Gorshkov, Tatyana Grigoryeva, Yurij Bukin, Anton Kuzmin

**Affiliations:** Limnological Institute, Siberian Branch of the Russian Academy of Sciences, 3 Ulan-Batorskaya Str., 664033 Irkutsk, Russia; krom_07@mail.ru (T.G.); bukinyura@mail.ru (Y.B.); kuzmin2000av@gmail.com (A.K.)

**Keywords:** priority PAEs, surface waters, concentration levels, anthropogenic and biogenic sources, environmental risk, Lake Baikal

## Abstract

Lake Baikal was studied as a model for elucidating the general pattern of *o*-phthalic acid diester (PAE) distributions in surface waters with background pollution levels. The influence of factors including congeners, concentrations, sampling points, seasons, years, and potential sources was considered and the environmental risk for various hydrobionts was established. Priority PAEs in Baikal waters are represented by dimethyl phthalate (DMP), diethyl phthalates (DEP), di-*n*-butyl phthalate (D*n*BP) and di-(2-ethylhexyl)phthalate (DEHP). Statistically valuable average concentrations and ranges for DMP, DEP, D*n*BP, and DEHP were 0.02 (0.01–0.02), 0.07 (0.06–0.09), 0.55 (0.47–0.66), and 0.30 (0.26–0.34) µg/L, respectively. The main factors determining PAE concentrations were the year and season of sampling, whereas sampling points were not among the factors influencing PAE levels. The distribution of PAEs in the water body was characterized by (i) an even distribution of minor hydrophilic DMP and DEP congeners in the whole water body, (ii) a maximum concentration of hydrophobic D*n*BP and DEHP congeners in the upper and near-bottom layers of the water column, and (iii) a low concentration of hydrophobic congeners in the near-shore area. The main PAE source was found to be the atmospheric transfer of polluted air masses, while the supply of PAEs from coastal sources to the pelagic zone was low. The contribution of biogenic sources to the background level of PAEs in the surface waters of Lake Baikal was established. The ecological risk of the background concentration level of PAEs for Lake Baikal biota was estimated. It was found that (i) DMP and DEP congeners do not represent a risk, or represent a very low risk, (ii) the concentration levels of dominant D*n*BP and DEHP congeners represent a low risk for crustaceans and fishes but (iii) a rather high risk for algae at a DEHP concentration of 0.30 µg/L.

## 1. Introduction

Ortho-phthalic acid diesters (PAEs) are some of the most important products of the chemical industry, and their global production volume reaches 6–8 million tons per year [1,2]. PAEs are used as plasticizers and added to polymer materials in order to obtain the required characteristics in plastics. The absence of chemical bonding between a polymer matrix and plasticizers results in the gradual migration of PAEs from plastic products into the environment during their exploitation and utilization. Stationary sources of PAEs (wastewater treatment plants of urban agglomerations, emission of industrial enterprises, areas of stock and treatment of industrial wastes and household plastics) determine a high content of these pollutants in the surface and underground waters of local sites. The number of PAEs can reach up to sixteen congeners, and their ratio and concentration depend on the sampling point and the presence of natural sources. In Lake Taihu (China), sixteen PAE congeners were found [3]. The range of PAE total concentrations (Ʃ_16_PAEs) was from 0.02 to 16 µg/L, and the dominant among them were D*n*BP, DEHP, and di-*i*-butyl phthalate (D*i*BP). In the water of the Kaveri River (India), six PAE congeners ranging in concentrations (Ʃ_6_PAEs) from 0.31 to 4.6 µg/L were established, and the dominant congeners were DEHP, DEP, and D*n*BP [4]. 

In background areas, traces of PAEs are detected in atmospheric aerosol and surrounding air, in surface waters, and in bottom sediments [5,6,7,8,9,10,11,12]. Atmospheric transport of polluted air masses and pollutant precipitation from the atmosphere are primary PAE sources in background areas, including waters in the central Arctic, the Norwegian coastal zone [13], and high mountains in China [14]. In the atmosphere, PAE congeners are distributed among aerosol particles and the gas phase of the surrounding air; the gas phase includes congeners containing short alkyl chains, while congeners with long ones are adsorbed in solid aerosol particles. From the atmosphere, PAEs accumulate on a spreading surface, whether land or water, through dry precipitation or through atmospheric precipitation of aerosol particles with adsorbed hydrophobic substances [6]. In surface waters with background levels of PAE pollution, D*n*BP and DEHP congeners are dominant, and changes in their source drastically influence both PAE compositions and total concentrations. 

PAEs are biologically active compounds and manifest hepo-, neuro-, and cytotoxicity. Toxic effects of PAEs, such as the disturbance of endocrine and reproductive functions, are found out not only in humans but also in various types of wildlife, such as mollusks, crustaceans, fishes, and invertebrates [15,16,17]. Taking into account the biological properties of PAEs and polymer material production volumes of up to 390 million tons per year [2], PAEs as industrial chemicals are included in the list of persistent organic pollutants (POPs) [18]. Among all PAEs, six congeners, namely dimethyl phthalate (DMP), diethyl phthalate (DEP), di-*n*-butyl phthalate (D*n*BP), benzyl butyl phthalate (BBP), di-(2-ethylhexyl) phthalate (DEHP), and di-*n*-octyl phthalate (DOP), are considered priority pollutants and are to be permanently monitored in the environment. 

Recent studies suggest a probable biosynthesis of PAEs by different plants, freshwater algae, cyanobacteria, and fungi [15]. PAEs are reported to possess allopathic activity and antimicrobial and insecticide properties, which increase the competitiveness of plants, algae, and microorganisms [19]. PAEs detected in surface water at trace levels are evidently related not just to anthropogenic pollutants, as they can accumulate from both abiogenic and biogenic sources, and the contributions of these two may be comparable in magnitude. We chose Lake Baikal (Russia) as a model for studies of the general pattern of PAE distributions in surface waters at background pollution levels. The chosen location is characterized by a huge water body volume, reaching up to 23,600 km^3^, a water surface area of 31,700 km^2^, a depth reaching up to 1642 m, the sharply continental climate of East Siberia, and the availability of potential POP sources on the shore. Lake Baikal is the largest natural reservoir of fresh water, containing up to 20% of the world’s freshwater reserves, and is characterized by a minimal content of suspended organic matter, a low degree of mineralization, and a background level of POPs [20,21,22,23]. To control PAEs in surface waters at background pollution levels, it is necessary to know the general pattern of their distribution.

The goals of the present study include (i) the identification and assessment of current PAE concentration levels in the waters of Lake Baikal, (ii) the determination of main factors influencing PAE concentrations, (iii) the analysis of PAE distributions in the water area and in deep horizons, (iv) the identification of PAE sources, and (v) the assessment of the environmental risk of PAEs in surface waters with a background level of pollution. In order to accomplish the aforementioned tasks, the long-term monitoring data of PAEs in the lake waters during the period from 2015 to 2022, particularly in the upper water layers of pelagic sites, deep horizons, the near-shore zone and bays of the lake, and the water of the south Baikal tributaries, were analyzed using a comparative statistical analysis.

## 2. Materials and Methods

### 2.1. Reagents and Instruments

The extraction of PAEs from water samples and the preparation of standard solutions for GC-MS analysis were carried out using *n*-hexane (HPLC grade, Cryochrom, Saint Petersburg, Russia) and acetone (reagent grade, EKOS-1, Old Kupavna, Russia). The extract aliquots were analyzed using Agilent 7890 B GC System 7000 C GC–MS Triple Quad equipment (Agilent Technologies, Santa Clara, CA, USA) and the HT-8 capillary column (30 m × 0.25 mm, 0.25 μm, SCE Analytical Science, Melbourne, Australia). The Phthalate Esters Mix EPA 606 M (Supelco, Darmstadt, Germany) and deuterated phthalates, dimethyl phthalate (DMP-d_4_), dipropyl phthalate (DPP-d_4_), and dihexyl phthalate (DHP-d_4_) (Witega, Berlin, Germany), were used as reference material and surrogate internal standards, respectively. The content of PAEs in organic solvents was controlled by a GC-MS prior analysis. The assessment of the ^13^C/^12^C ratio in PAEs from water samples was performed using HPLC-ESI-HRMS-TOF equipment. The concentration step was carried out using the C_18_ reversed phase column (75 × 2 mm, Nucleosil 100-5-C18, Macherey-Nagel, Duren, Germany) at a flow rate of 0.2 mL/min for 50 min. Phthalates were eluted and detected using high-resolution time-of-fight mass spectrometer (Agilent 6210, Agilent, USA) in the ESI+ mode; the mass range *m/z* from 60 to 600; the carrier gas (nitrogen, 99.8%) flow and temperature were set at 5 L/min and 325 °C, respectively; the nozzle pressure was 45 psi and the capillary voltage was 3.5 kV. The device calibration was performed using the ESI-L Low Concentration Tuning Mix (P/N G1969-85000, Agilent, Santa Clara, USA) monthly.

### 2.2. Water Sampling

**Figure 1:**
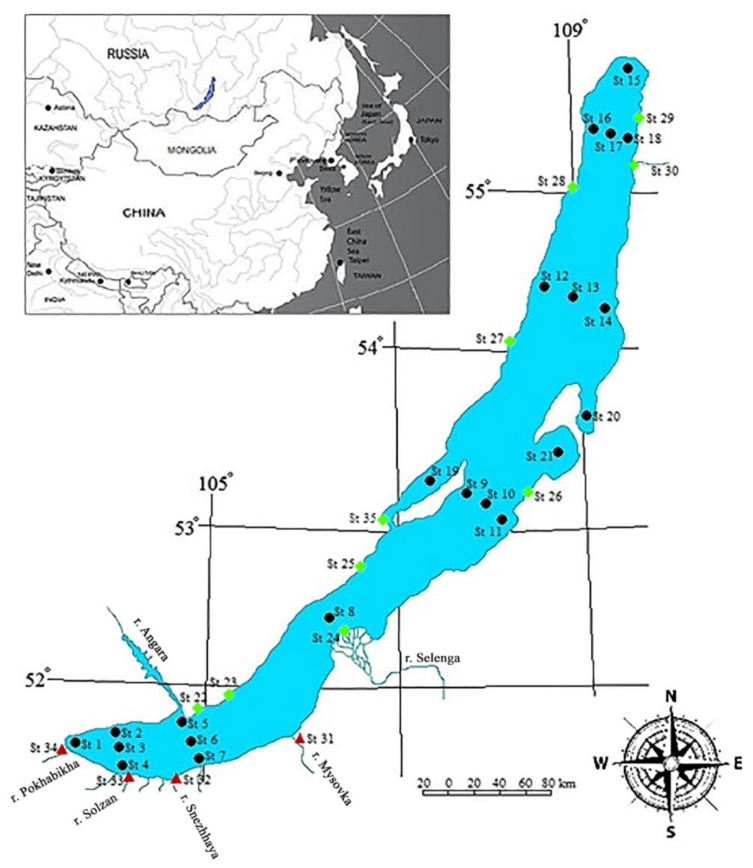
Map of Lake Baikal and sampling points. 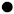
— Pelagic zone. At Stations 1, 2, 4, 5, 7, 8, 9, 11, 12, 14, 15, 16, and 18, sampling was carried out from the upper water layer (5 m) at a distance of up to 3 km from the coast; Stations 3, 6, 8, 10, 13, and 17 are reference central stations; at Station 6, samples were taken from the water column at depths from 5 to 1200 m; at Station 10—from the water column at depths from 5 to 1600 m; at Station 17—from the water column at depths of 5 to 800 m; Stations 2–4, the section of Maritui settl.—Solzan River; Stations 5–7, the section of Listvyanka settl.–Tankhoy settl.; Stations 9–11, the section of Ukhan cape–Tonkii cape; Stations 12–14, the section of Elokhin cape–Davsha settl.; Stations 16-18, the section of Baikalskoe settl.–Turali cape. 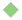
— Coastal zone, Stations 22–30, 35; 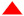
—River mouths, Stations 31 (the Mysovka river, Babushkin settl.), 32 (the Snezhnaya river), 33 (the Solzan river), 34 (the Pokhabikha river, Sludyanka City).

Water samples were taken from under ice (March) after the disappearance of the ice cover on the lake (late May–early June) and in the beginning of the autumn season (September) during the period from 2015 to 2022. The water samples from pelagic site and deep horizons of the lake (Figure 1) were collected using the SBE-32 cassette sampler (Carousel Water Sampler, Sea-Bird Electronics, Bellevue, WA, USA). The water samples from Baikal tributaries were taken from the upper river mouth layers (0 m). In the near-shore zone, the samples were collected from the upper water layer (0 m) at a distance of more than 50 m from the shore and at a depth of no more than 20 m. At each station, two water samples of ca. 1 L each were bottled into amber glass tare, and 0.5 mL of a 1 M aqueous solution of sodium azide (Merck, Germany) was added as a preservative. The bottles were closed using a lid with the aluminium foil gasket and stored at 5 °C before laboratory analysis.

### 2.3. Sample Processing

In order to decrease the number of sample preparation stages and to assess the contribution of biota, non-filtered water samples were analyzed. Deuterated phthalates were added to water samples prior to extraction and quantification of analytes by means of GC-MC. The content of PAEs in water samples was estimated by approach described in [24] including liquid–liquid extraction of PAEs by *n*-hexane. For analysis, an aliquot was taken from the extract (single extraction) and analyzed by GC-MS. The temperature program for the GC oven was started from 50 °C and then increased up to 300 °C at the rate of 25 °C/min, and finally held for 5 min. The sample injector temperature was set up at 280 °C, and 2.0 μL of the sample solution was injected using the splitless mode. The ion source and quadrupole temperatures were maintained at 230 °C and 150 °C, respectively. The electron impact ionization energy was 70 eV. An ultra-pure helium (99.999%) at a constant pressure of 25 psi was used as a carrier gas. PAE peaks were recorded using the SIM mode and the reference ions *m*/*z* 149, 153, 163, 167. The peak assignment was performed by comparing their retention times with those of standard solution. PAE congeners were quantitated using the method of internal standards. The GC-MS device was calibrated in the range of expected PAEs concentrations from 0.01 to 10 µg/L. The reliability of approximation of calibration dependences S_a_/S_st_ = k(m_a_/m_st_) corresponded to the condition R^2^ ≥ 0.99. The concentrations of PAE in water samples were calculated as the average value of two parallel samples. The secondary contamination of the analyzed samples by PAEs from the laboratory background was evaluated by the procedure of blank experiment and subtracted accordingly. The PAE limit of determination was estimated as 0.008–0.20 µg/L, and the relative standard deviation (RSD^Rl^) was 7.0–20% for individual congeners.

The ratio of stable carbon isotopes ^13^C/^12^C in the composition of DEHP from surface waters was estimated using an approach proposed by us earlier [25]. Briefly, the ^13^C/^12^C ratio in DEHP is calculated as a ratio of the peak areas of the monoisotopic masses [M + 1 + H]^+^ and [M + H]^+^. The value of Δ^13^C (should not be compared with *δ*^13^C measured according to the international VPDB scale) in DEHP is estimated relatively to the ^13^C/^12^C ratio in the commercial DEHP standard. The values of Δ^13^C in DEHP of tentatively biogenic and anthropogenic sources were used as the lower and upper frontier limits on the Δ^13^C scale, respectively. The DEHP from Baikal phytoplankton (Δ^13^C = −46‰) and cell culture of *Aconitum baicalense* Turcz ex Rapaics 1907 (Δ^13^C = −50‰) was assigned as an isotopologue of biogenic origin. The DEHP isotopologue from just fallen snow on an urban territory (Δ^13^C = +5.2‰) and that from treated sewage water (Δ^13^C = +0.2‰) were assigned as the congeners from an anthropogenic source. The minimal concentration of DEHP in water required for reliable determination of ^13^C/^12^C value was estimated at the level of ca. 0.2 µg/L. 

The PAE content in phytoplankton biomass samples was assessed using the method described in [25], which includes the use of an Apstein net (cell diameter 30 μm) for sampling, further samples drying at room temperature, homogenization and double extraction by 10 mL of a *n*-hexane–acetone (1:1, by volume) mixture followed by GC-MS and HPLC-HRMS-TOF analyses of 1 mL aliquots.

### 2.4. Environmental Risk Assessment

According to the European technical guidance document on risk assessment [26], the risk coefficients (RQ) were calculated using the average concentration of PAE congeners (C_PAE_, µg/L) and the predicted no effect concentration (PNEC, µg/L); Equation (1):

RQ = C_PAE_/PNEC. (1)

In the present work, the PAE concentrations measured in Baikal biota [27] and those from [28] were selected as PNEC values (refer to Table S2). 

### 2.5. Statistical Methods

Pairwise correlations between two replicates of measurements and the average concentration of PAEs over the entire concentration range for priority congeners in samples of Baikal water were estimated with Spearman’s r correlation coefficient. *p* values for the correlation coefficients were calculated using Spearman’s “W” statistics. Unreliable values of correlation coefficients (*p* values > 0.05) were replaced with 0 values. Pairwise correlations were visualized with a heat map generated using “gplots” in [29]. Lines and columns in the correlation matrix were clustered and grouped in order of similarity (i.e., the Euclidean distance metric and the complete-link clustering method).

For further analysis, these missing data were replaced with averages for these congeners according to recommendations [30]. Water samples that contained concentrations of congeners (at least one congener) outside the limits of three differences between the first (Q1) and third (Q3) quartiles from the median concentration values were considered outliers and excluded from the analysis. PERMANOVA uses the Euclidean distance metric and 1000 permutations for *p* value calculations. Before PERMANOVA analysis, all concentration values were transformed to eliminate the physical dimensions by ranging from 0 to 1.

Confidence intervals for the mean values of PAE concentrations, grouped according to the explanatory parameter (year, season (spring, autumn), ecotope (pelagic, coastal, bay, river, sampling site (pelagic or other), basin (southern, central, northern), sample points), were estimated using the bootstrap method in the “boot” package and the R programming language (1000 bootstrap replicas).

## 3. Results and Discussion

### 3.1. Identification and Assessment of Current PAE Concentration Level in Lake Baikal Waters

**Figure 2:**
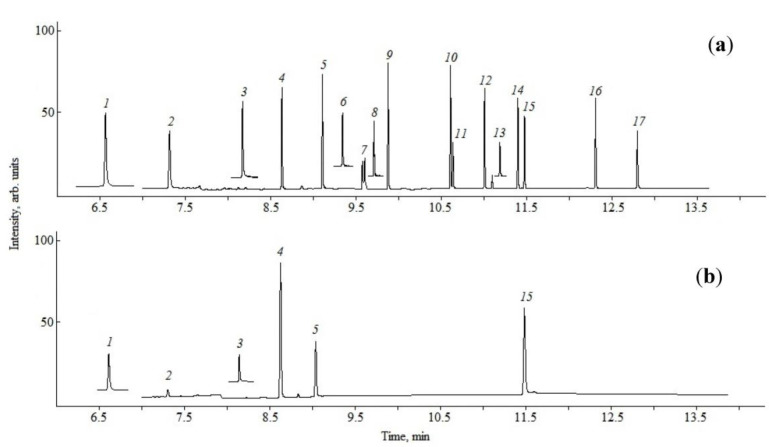
Mass fragmentograms: (**a**) Analyte Mix #3 (Dr. Ehrenstorfer Reference Material); (**b**) Extract of the upper water layer of Lake Baikal (5 m). Peak Nos. (*m*/*z*): 1—DMP (163); 2—DEP (149); 3—benzoic acid–benzyl ester (105); 4—D*i*BP (149); 5—D*n*BP (149); 6—dimethyl glycol ester (59); 7—bis-4-methyl-2-pentyl ester, two conformers (149); 8—bis-2-ethoxyethyl ester (72); 9—bis-*n*-pentyl ester (149); 10—bis-hexyl ester (149); 11—BBP (149); 12—hexyl-2-ethylhexyl ester (149); 13—bis-2-*n*-butoxyethyl ester (57); 14—bis-cyclohexyl ester (149); 15—DEHP (149); 16—DOP (149); 17—bis-nonyl phthalate (149).

In Lake Baikal waters (pelagic sites, deep horizons, near-shore zones, Chivyrkuisky and Barguzinsky Bays, and the Maloye Morye, Figure 1), five PAE congeners, namely DMP, DEP, D*i*BP, D*n*BP and DEHP, were found. All of these congeners except D*i*BP are among priority substances in accordance with the system of POPs control in surface waters. The DOP congener from the priority list was not detected in Baikal waters at the level of >0.01 µg/L (Figure 2).

**Figure 3:**
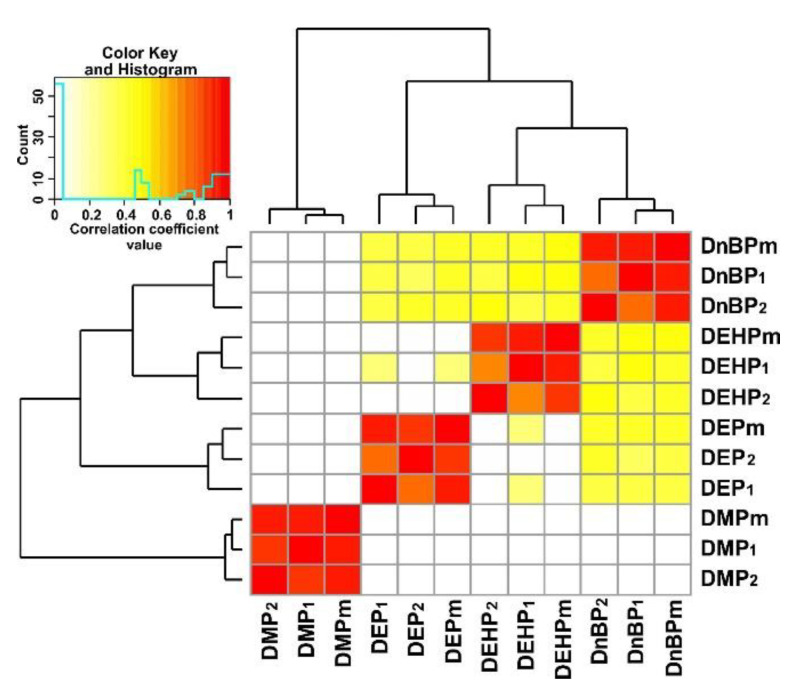
Heatmaps reflecting values of correlation coefficients between the results of PAE concentration determination in parallel samples (PAE_1_, PAE_2_) collected at the same station and calculated average value (PAE_m_).

Concentration levels of priority PAE in Lake Baikal were assessed as following statistically valuable average values and concentration ranges, µg/L: 0.02 (0.01–0.02), 0.07 (0.06–0.09), 0.55 (0.47–0.66), 0.30 (0.26–0.34) for DMP, DEP, D*n*BP and DEHP, respectively. The dominant contribution of both D*n*BP and DEHP congeners of up to 90% into total PAE content (Ʃ_4_PAEs) was observed (Table S1). The data of correlational analysis (Figure 3) confirmed a high convergence between results of PAE determination in two parallel samples collected at the same station. Therefore, a subsequent analysis of monitoring data was performed using average values of PAE concentrations. It is worth noting that there was no statistically valuable correlation between concentrations of DMP congener and other priority PAEs. In contrast, moderately positive correlational links between concentrations of DEP, D*n*BP and DEHP (*p* value from 0.47 to 0.51) were observed.

### 3.2. Main Factors Affecting PAE Concentration Levels

The analysis of PAE monitoring results in water samples of Lake Baikal using the PERMANOVA approach showed that the concentration of this group of POPs is reliably related to a sampling year (the most valuable factor among considered ones) and to sampling season, spring or autumn (second factor by its validity), whereas sample points and basin are not among the factors influencing PAE concentration levels (Table 1).

**Figure 4:**
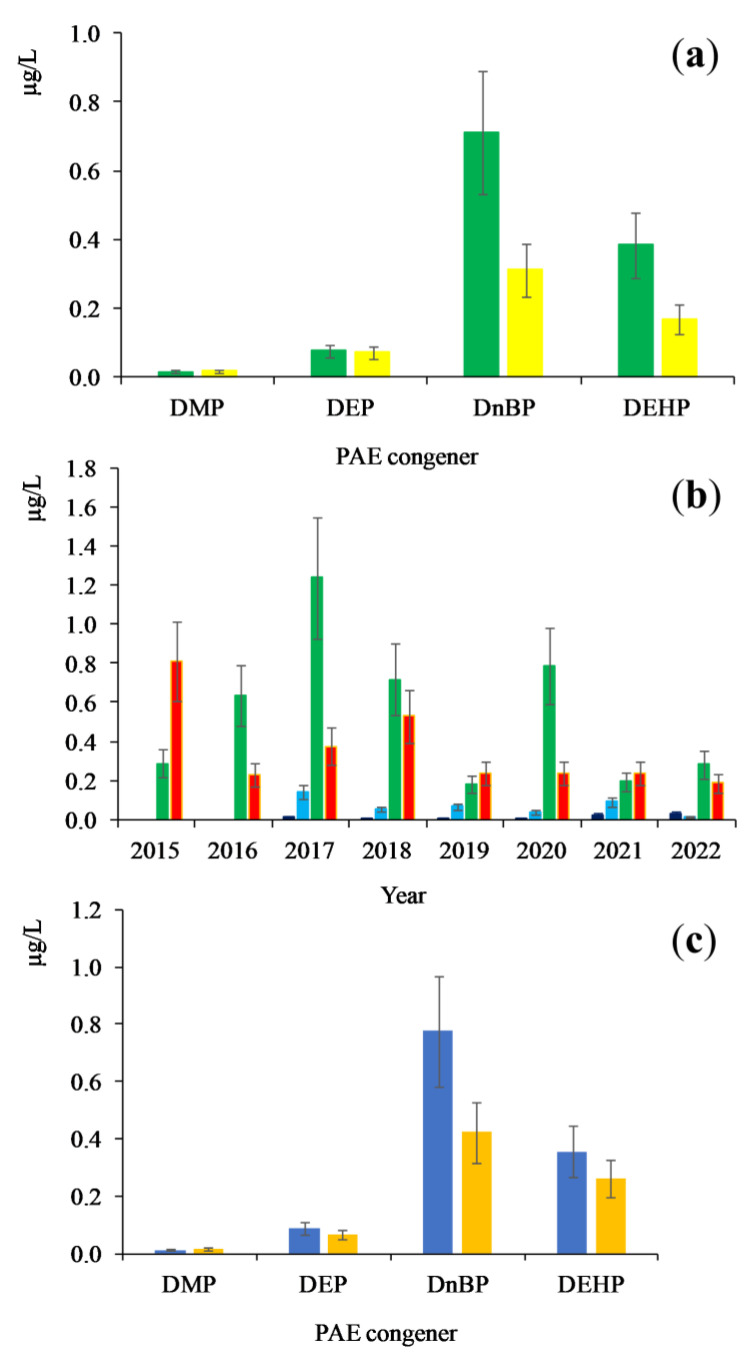
Average concentrations of priority PAEs in Lake Baikal waters during the monitoring period of 2015–2022. (**a**)—Seasonal distribution of concentrations of PAEs: 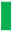
—spring, 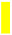
—autumn; (**b**)—Interannual distribution of concentrations of PAEs: 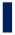
—DMP; 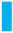
—DEP; 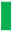
—DnBP; 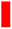
—DEHP; (**c**)—Distribution of concentrations of PAEs: 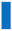
—central zone; 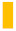
—other areas.

Average concentrations of both DMP and DEP congeners were low and practically did not depend on a season and on the lake basin. In contrast, the concentrations of D*n*BP and DEHP congeners were higher by one order of magnitude. Their seasonal variability is drastic and individual for different monitoring years (Figure 4a,b). Correlational links between concentrations of priority PAEs are absent or moderately positive (Figure 3). For example, the increase of DMP congener concentration was detected in 2021 and 2022, DEP congener in 2017, while maximum concentration level of D*n*BP congener was observed in 2017 and 2020 and that of DEHP congener in 2015 and 2018.

The analysis of PAE monitoring data (Table 1 and Figure 4c) revealed the division of sampling points in two groups: (i) the central zone of the pelagic section of the lake (Stations 3, 6, 8, 10, 13, 17), and (ii) the coastal zone, large bays and river mouths. Both of them significantly influence the range of PAE concentrations. Therefore, one can conclude that the PAE concentrations in the central section of the pelagic zone of Lake Baikal vary irrespective of the PAE concentrations in other ecotopes of the coastal zone. The concentrations in the central zone of the pelagic area are not influenced by local coastal sources of PAEs. Thus, the PAE concentrations in the central zone of the lake characterize their level in the lake, which can be represented by the statistically significant range and average value. This conclusion is very important for organizing a PAE monitoring system.

### 3.3. Distribution of PAEs in Water Area and in Deep Horizons of Lake Baikal

Along the water area and deep horizons, PAEs are distributed according to their hydrophobicity. The constant log*K_ow_* for priority congeners is estimated within the range from 1.60 to 8.10 [31]. DMP and DEP congeners are characterized by high hydrophilicity and are evenly distributed in the whole water body in the range of average concentrations from 0.01 to 0.09 µg/L. Hydrophobic congeners are adsorbed on suspended particles presented in water column and are transferred into bottom sediments. Due to this fact, in the near-shore zone, which is characterized by a high content of suspended particles, intensive stirring and thinner water layer, a lower average concentrations of both D*n*BP and DEHP congeners than in the central zone of the pelagic site are found (Figure 4c). The concentrations of these congeners in the central zone are assessed by statistically valuable average concentrations equal to 0.78 and 0.36 µg/L, respectively, which are up to two times higher compared with that in the near-shore zone (Table S1). This result is rather unexpected taking into account PAE accumulation from sources located on the shore and, consequently, an increase in PAE concentrations at some near-shore sites is expected. For example, at Station 29 (2017, spring) and Station 28 (2017, autumn), anomalously high concentrations of Ʃ_4_PAEs, 7.4–9.0 µg/L, were observed. On the other hand, the results of statistical analysis indicate that lower values of the average concentrations of both D*n*BP and DEHP congeners in the coastal zone did not affect PAE levels in the central zone of the lake.

**Figure 5:**
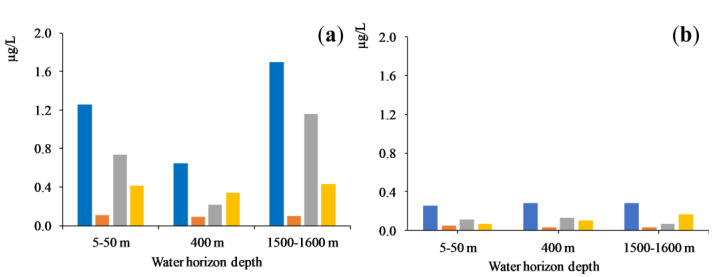
Mean concentrations of PAEs at deep horizons of Central Baikal water column, Station 10: (**a**) spring; (**b**) autumn. 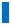
—Ʃ_4_PAEs, 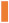
—DEP, 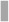
—D*n*BP, 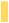
—DEHP.

The distribution of PAEs in the water column is perfectly reflected by the concentrations of congeners at central basin stations of Lake Baikal at a depth of up to 1600 m (Figure 5). During the spring season, the highest concentrations (Ʃ_4_PAEs) were detected in upper and near-bottom water layers with the predominant contribution of hydrophobic D*n*BP and DEHP congeners. As a result of biodegradation and transfer of D*n*BP and DEHP congeners into bottom sediments during the summer season, the total concentration (Ʃ_4_PAEs) was decreased by three- to fourfold. In the autumn season, the concentration of priority PAEs did not depend on water sampling depth and reflected a background level of water pollution by phthalates.

As noted above, the concentration of PAEs in the water of the coastal zone was not influenced by its level in the pelagic zone of the lake. Nevertheless, in case of large bays, where recreational areas are located on the shore, a rather high level of PAEs was observed. For example, in the water of the Maloye Morye (Station 19), priority congeners were found in concentration range from 0.58 to 1.8 µg/L (Ʃ_4_PAEs), the latter value up to three times higher than an average concentration of Ʃ_4_PAEs in the lake waters. At Chivyrkuy Bay (Station 20), no dependence between the Ʃ_4_PAE average concentration and season (0.82–0.77 µg/L, spring–autumn) was found, while at Barguzinsky Bay (St. 21) as well as in case of the pelagic sites of the central basin (St. 21, 10), the concentration of Ʃ_4_PAEs was characterized by a threefold decrease in autumn.

### 3.4. Origin of PAEs in the Water of Lake Baikal

#### 3.4.1. Anthropogenic Sources

One of the main sources of PAEs is attributed to atmospheric transport of air masses contaminated by these compounds and their accumulation in the surface layer of water along with aerosol particles on which hydrophobic compounds were initially adsorbed. The dominance of hydrophobic D*n*BP and DEHP congeners in the upper water layer of the pelagic zone of the lake confirms this conclusion. During the winter season, PAEs from the atmosphere are accumulated in the ice as well as in the snow cover on the shore. It results in the migration of PAEs into the lake in spring along with melt waters (total concentration of priority PAEs in the snow cover of the southern shore of Lake Baikal were in the range from 0.46 to 3.9 µg/L). Such a source of PAEs is obviously referred as the one determining the sharp seasonal variability of D*n*BD and DEHP levels. The monitoring of PAEs in the water of south Baikal tributaries, having water drainage basins on the territory of natural reserves, confirms this assumption.

The level of Ʃ_4_PAEs increases in the water of lake tributaries in the spring season, sometimes by one order of magnitude compared with that found in autumn. Average concentration of Ʃ_4_PAEs in the mouth of tributaries was equal to 0.79 µg/L and in the range from 0.56 to 1.1 µg/L (autumn–spring). It is noteworthy that the contribution of both D*n*BP and DEHP congeners in the water of tributaries is comparable (the D*n*BP/DEHP ratio is in the range from 0.99 to 1.1). In the central zone of the lake, the ratio is equal to 2.2, indicating the migration of a part of PAEs from tributary watersides to the former. 

Thus, a potential anthropogenic source of PAEs may be attributed to the lake tributaries, the water of which may contain treated sewage water from wastewater treatment plants of cities located on the shore. For example, the concentration of Ʃ_4_PAEs in the wastewaters from Slyudyanka City purification facilities is within the range from 21 to 29 µg/L, and the content of DMP and DEP congeners is 20 and 46%, respectively. In the water from the mouth of the Pokhabikha River (Slyudyanka City, Station 34) and the Mysovka River (Babushkin settlement, Station 31), the concentrations of Ʃ_4_PAEs did not exceed 2.6–3.0 µg/L, while the content of hydrophilic DMP and DEP congeners was up to 7–20% of the total concentration of Ʃ_4_PAEs, respectively. In the Selenga River, shallow water area (a part of Lake Baikal, Station 24) opposite to the river delta with a depth ≤ 400 m, the concentration of Ʃ_4_PAEs did not exceed 0.77 µg/L (Station 24, 2021, autumn). The content of the DEP congener was high and equal to 20% of Ʃ_4_PAEs, evidently due to the phthalate accumulation along with wastewaters drained into the Selenga River. 

Besides wastewaters, household wastes on the shore consisting mainly of plastics (bottles, containers, package material, etc.) are also related to the anthropogenic source of PAEs. In water samples from the littoral close to the recreation center (Station 28, 2017, September) the concentration of Ʃ_4_PAEs was equal to 7.4 µg/L, and the dominant congener was DEP (78% of Ʃ_4_PAEs). It is worth noting that a high concentration of DEP occurs as a result of biodegradation of PAEs containing long alkyl groups [32] or due to direct accumulation from cosmetic packages and personal hygiene products [33]. The pollution of water bodies by household plastics is considered as one of the probable PAE sources in aquatic environment. Previous model experiments showed [34] that colonies of microorganisms on plastic surfaces may recycle PAEs in water bodies. Due to the fact that the rates of PAE diffusion and biodegradation are comparable, only a fraction of phthalates enters into the water. 

#### 3.4.2. Biogenic Sources of PAEs

The atmospheric transport of polluted air masses provides the way for anthropogenic PAEs entering into water of Lake Baikal. When the lake is ice covered, the atmospheric transport of PAEs is rather limited and cannot influence the composition of PAEs under the ice. In water samples taken from under the ice, wide variations in PAE composition are observed. The concentrations of Ʃ_4_PAEs in the range from 1.8 to 6.1 µg/L were found. The contribution of DMP, DEP and D*n*BP congeners into it did not exceed 0.33 µg/L, while extreme levels of DEHP congener of up to 5.8 µg/L were found. 

**Figure 6:**
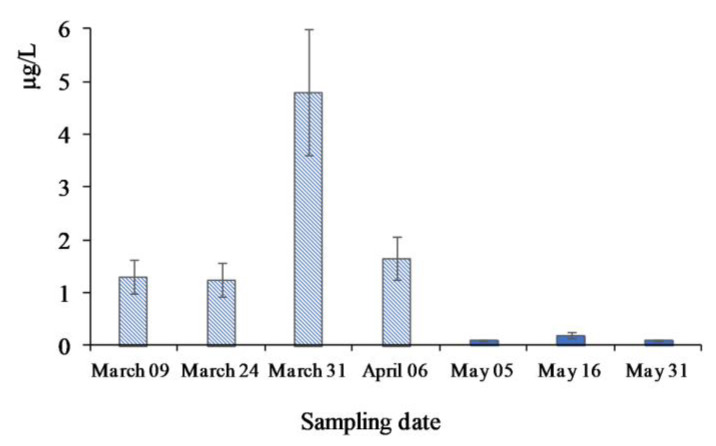
DEHP concentration in the water of the coastal zone of Lake Baikal. Sampling from under the ice (March–April) and from open water (May).

The increase in DEHP concentration in under-ice water samples was also detected during PAE monitoring in the coastal zone of the lake (Figure 6) in March–May. Sampling there was performed from under the ice and from open water [24]. Such a change in PAE composition could be associated with the alteration of a dominant source. The possibility of phtalate biosynthesis implies the presence of a biogenic source, in particular for PAEs found in the waters of Lake Baikal. Detected concentrations of DEHP in the water during the spring season may be associated with under-ice diatom algae blooms. Indeed, phytoplankton collected along with water samples contained 60–170 µg/g (dry biomass) of DEHP. The main component of phytoplankton was identified as freshwater diatom *Synedra acus* subsp. Radians (Küts), which could be a potential source of DEHP during the spring period. The value of stable carbon isotope ^13^C/^12^C (Δ^13^C) ratio in DEHP from under-ice water samples confirmed substantial contribution from a biogenic source to this congener (Δ^13^C of ca.–24‰).

The ^13^C^3^/^12^C (Δ^13^C) ratio in DEHP allowed drawing a conclusion on the contribution of a biogenic source into the background PAE levels in Lake Baikal surface waters. In the southern basin of the lake (central stations, Stations 3, 6), the Δ^13^C ratio was estimated at −16 and −10 ‰, respectively, while in the northern basin, it was at −30 ‰ (Station 17). The greater contribution of anthropogenic PAEs to the basin is obviously associated with POP atmospheric transport from the industrial zone along the Angara River [35]. In the northern basin, ice melting occurs somewhat later as well as the diatom growth compared with that in the southern one. This fact determines a greater contribution of PAEs from the biogenic source to their total content found in the northern part of the lake. Water samples collected from the littoral part of the lake (Stations 22, 35—popular recreation sites) contained 0.32 and 0.78 µg/L of Ʃ_4_PAEs; the DEHP congener was dominant. At Station 22, the Δ^13^C value for the congener was equal to −37‰, indicating a high contribution of a biogenic source, while at Station 35, the Δ^13^C value was only −10‰, and DEHP was of a rather anthropogenic origin. In water samples from lake tributaries, the Snezhnaya River and the Solzan River (Stations 32 and 33), concentrations of Ʃ_4_PAEs were 0.31 and 0.43 µg/L, respectively; DEHP was dominant. The measured Δ^13^C ratio of the latter showed the presence of a notable isotopologue contribution from a biogenic source (Δ^13^C, −20‰) in samples from the Solzan River, while a large DEHP content of anthropogenic origin (Δ^13^C, 5.6‰) in samples from the Snezhnaya River was found. Taking into account a huge drainage basin of the Snezhnaya River of ca. 3,000 km^2^, the transport of POPs from the atmosphere to the water in there may saturate the latter by anthropogenic PAEs resulting in increased Δ^13^C values.

### 3.5. Ecological Rick Assessment of PAEs in Surface Waters

The concentration of priority PAEs in Baikal waters (0.26–0.34 µg/L) is comparable to or less than those found in other freshwater lakes and rivers worldwide (Table 2). The level of the DEHP congener in Lake Baikal is more than one order of magnitude lower than maximum permissible concentration (MPC): 8 µg/L for drinking water [36], 1.3 µg/L for fresh and marine waters [37], 8 µg/L for Russian water bodies [38]. The DEHP concentrations of up to 5.8 μg/L observed under the ice are alarming, as they are close to the MPC level. A potential environmental risk for Baikal biota from PAEs was assessed by RQ coefficients calculated using average concentrations of priority PAEs in waters (Table S2). 

The DMP and DEP congeners found in pelagic area of the lake do not represent or represent a very low risk for hydrobionts such as fishes, crustaceans and algae due to calculated RQ values being <0.01. The concentration of dominant D*n*BP and DEHP congeners represent a low risk level for crustaceans and fishes and a high one for algae at the DEHP concentration of 0.30 µg/L. Thus, the increase in the latter content in the pelagic site of the lake in the spring season and at some sites of the near-shore zone as a result of accumulation of hydrophobic congeners in deep horizons drastically increases the environmental risk to average or even high one for algae (Table S3). The presence of D*n*BP congener in the water of tributaries is associated with a very low risk level, while DEHP is associated with a moderate to high one. 

The extreme level of DEHP in under-ice water was estimated by the RQ coefficient ˃˃ 1.0. At the same time, the measured stable carbon isotopes ^13^C/^12^C ratio in DEHP explains the phenomenon of its high concentration originated from a potential biotic source. It should be noted that (i) DEHP congener is a potential carcinogenic compound [39] and its MPC is high, equal to 8.0 µg/L; (ii) structurally, DEHP is presented by two stereoisomers, the only one of which, namely bis-2R(-)ethylhexyl phthalate, has been isolated from environmental objects so far; for example, the one isolated from the cells of the *Aconitum baicalense* (Turcz ex Rapaics 1907) culture and from brown sugar [40,41]; (iii) DEHP toxicological studies were conducted using a synthetic racemic product, while the natural DEHP stereoisomer does not have to obligatory possess the same physiological properties [42]; (iv) while obtaining extreme DEHP concentrations, it is necessary to identify the source which could be biogenic or anthropogenic, one or both, before taking any environment protections. 

**Table 2:** Concentrations of PAEs in surface water of recent studies, µg/L.

Area	DMP	DEP	D*n*BP	BBP	DEHP	D*n*OP	ƩPAEs	Reference
Lakes in Hanoi metropolitan area, Vietnam	0.11–2.9	0.64–14	0.78–34	0.18–21	1.0–49	˂0.02–7.3	Ʃ_10_ 19–130	[43]
Lake Victoria, Uganda	0.006–0.40	0.04–1.1	0.35–16	-	0.21–23	–	Ʃ_4_ 0.67–50	[44]
Yangtze River, China	˂0.01	˂0.01	0.22–20	˂0.01–0.02	0.02–7.0	˂0.01	Ʃ_16_ 0.44–20	[45]
Lake Taihu, China	˂0.02–0.80	˂0.02–0.12	˂0.02–0.19	˂0.02–1.3	˂0.02–3.3	˂0.02–0.65	Ʃ_16_ 0.02–16	[3]
Kaveri River, India	˂0.01–0.01	0.04–0.52	˂0.01–0.37	˂0.01–0.14	˂0.01–0.82	˂0.01–0.08	Ʃ_6_ 0.04–4.6	[4]
Lake Large Xingkai, China	0.003–0.026	0.003–0.018	0.11–0.52	0.11–0.52	0.22–3.4	nd –0.007	Ʃ_8_ 0.35–3.8	[46]
Lake Asan, Korea	˂0.02–0.18	˂0.02–0.05	˂0.02–0.34	˂0.02	˂0.02–1.3	˂0.02–0.02	Ʃ_14_ 0.02–1.9	[47]
Lakes in Summer Palace, China	0.04–0.08	˂0.01–0.01	0.03–0.04	˂0.01–0.01	0.14–0.39	˂0.01–0.02	Ʃ_15_ 0.58 –1.4	[48]
Changjiang River Estuary, China	0.04–0.28	0.02–0.18	0.03–2.4	˂0.01	˂0.01–0.01	˂0.01–0.01	Ʃ_16_ 0.27–1.3	[49]
Mediterranean Sea, Bay of Marseilles, France	˂0.01	˂0.01–0.05	0.06–0.46	˂0.01	0.10–0.30	˂0.01	Ʃ_8_ 0.24–1.2	[11]
Lake Baikal, Russia	0.01–0.02	0.06–0.08	0.47–0.66	˂0.01	0.26–0.34	˂0.01	Ʃ_4_ 0.66–0.87 ^1^	This study

Note: ^1^—for Lake Baikal waters the mean value of Ʃ_4_PAEs is assessed as 0.76 µg/L and confidence interval of 0.66–0.87 µg/L (*p* = 0.95).

In the environment, PAE congeners are slowly degraded by various physico-chemical processes. In particular, the half-life value (t½) of phthalates under hydrolysis conditions reaches more than 100 years. Biodegradation is considered as the most important process of PAE removal from water. Under aerobic conditions, it occurs with a high rate, and the t½ value is from 1 to 14 days [6,34,50]. The decrease in the content of PAEs in surface waters of Lake Baikal in the summer season is obviously associated both with the degradation of PAEs entering the lake in the spring and with the decrease in the number of their sources. Thus, a dynamic equilibrium between PAE accumulation from different sources, biodegradation processes and the transfer of dominant hydrophobic congeners into bottom sediments is established, and it is the one determining the background level of PAEs in Lake Baikal.

## 4. Conclusions

Lake Baikal as a model of surface waters with a background level of pollution was studied. The waters of the lake contain only four priority phtalates, PAEs, namely DEP, DMP, D*n*BP, and DEHP at trace concentration levels. Statistically valuable average concentrations and ranges of PAEs were 0.02 (0.01–0.02), 0.07 (0.06–0.09), 0.55 (0.47–0.66), 0.30 (0.26–0.34) µg/L for DMP, DEP, D*n*BP, and DEHP, respectively. The dominant contribution of D*n*BP and DEHP congeners into the total PAE concentration was up to 90%. The main factors determining PAE concentrations were the year and season of sampling, whereas sampling points were not among the factors influencing PAE levels. Under the conditions of sharply continental climate, the PAE concentrations are characterized by seasonal variability. They are increasing in the spring and decreasing to the background levels by the end of summer. One of the main sources of PAEs to the waters of the lake is atmospheric transport of air masses contaminated by POPs along with aerosol particles. The distribution of PAEs in the water depends on their hydrophobicity. The minor hydrophilic DMP and DEP congeners are distributed throughout both the water area and the water column; hydrophobic D*n*BP and DEHP congeners are accumulated in the upper and near-bottom layers of the water column. The contribution of PAEs from coastal sources to the pelagic zone is low due to the rapid transport of hydrophobic congeners into coastal sediments. The ecological risk for the Baikal biota does not represent or represents a very low level in case of DMP and DEP congeners, while the dominant D*n*BP and DEHP congeners possess a low risk level for crustaceans and fishes and a high one for algae at DEHP concentrations of 0.30 μg/L. A confirmation of the contribution of biogenic sources to the background level of PAEs in the surface waters of Lake Baikal was also determined. When exploring the extreme concentrations of DEHP, it is highly necessary to identify its source (biogenic or anthropogenic) prior to drawing any conclusions. 

## Figures and Tables

**Table 1:** Results of PERMANOVA statistical analysis of PAE monitoring data in Lake Baikal *.

Factor	R^2^ Value	*p* Value
Year (2015–2022)	0.0428	0.0009
Season (spring, autumn)	0.0348	0.0009
Ecotope (pelagic, coastal, bay, river)	0.0237	0.0049
Sampling site (central zone or other)	0.0224	0.0009
Basin (southern, central, northern)	0.0067	0.2297
Sample points	0.0024	0.2437

* The greater the R^2^ value, the greater the influence of the factor on the distribution of PAE concentration. If *p* value ≤ 0.05, the factor influences reliably the level of PAE concentrations in Lake Baikal water.

## Data Availability

The datasets obtained and analyzed in the current study are available from the corresponding author on reasonable request.

## Supplementary Materials

The following supporting information can be downloaded at: https://www.mdpi.com/article/10.3390/toxics11100869/s1, Table S1: Average values (confidence intervals, *p* = 0.95) for different groups of results of PAE monitoring in Lake Baikal waters. Table S2: Assessment of environmental risk for hydrobionts by average concentrations of priority congeners PAEs. Table S3: Assessment of environmental risk for dominant congeners of PAEs at high concentration levels (C, µg/L) in waters of Lake Baikal and in tributary mouths.
