# Supplementary material for: Case Study of Diesters of o-Phthalic Acid in Surface Waters with Background Levels of Pollution
Source: Toxics. 2023 Oct 19;11(10):869. doi: 10.3390/toxics11100869 (PMC10611372; doi:10.3390/toxics11100869)
Supplement: Supplementary file 1 [file toxics-11-00869-s001.zip › toxics-2590620-supplementary.pdf]

Table S1. Average values concentrations for different groups PAEs monitoring results in Lake Baikal waters (confidence intervals, P=0.95). The confidence intervals are calculated using boot strap method

Data groups	PAEs, µg/L			
	DMP	DEP	DBP	DEHP
Lake Baikal waters	0.016 (0.015 0.020)	0.073 (0.063 0.087)	0.548 (0.474 0.662)	0.296 (0.261 0.338)
Seasons				
Spring	0.015 (0.012 0.020)	0.075 (0.061 0.094)	0.712 (0.597 0.893)	0.383 (0.337 0.452)
Autumn	0.018 (0.015 0.0223)	0.071 (0.057 0.092)	0.311 (0.251 0.410)	0.169 (0.141 0.210)
Basins				
Southern	0.02 (0.0157 0.0266)	0.0726 (0.0603 0.088)	0.493 (0.41 0.586)	0.271 (0.233 0.322)
Central	0.012 (0.009 0.015)	0.062 (0.047 0.088)	0.688 (0.506 1.15)	0.311 (0.250 0.405)
Northern	0.014 (0.011 0.018)	0.0835 (0.060 0.118)	0.521 (0.396 0.765)	0.322 (0.251 0.418)
Year				
2015	-	-	0.288 (0.215 – 0.429)	0.809 (0.577 – 1.12)
2016	-	-	0.636 (0.511 – 0.792)	0.231 (0.165 – 0.35)
2017	0.013 (0.009 – 0.016)	0.145 (0.116 – 0.185)	1.24 (0.944 – 1.86)	0.377 (0.302 – 0.474)
2018	0.001 (0.001 – 0.003)	0.056 (0.048 – 0.068)	0.719 (0.563 – 0.889)	0.529 (0.376 – 0.842)
2019	0.006 (0.004 – 0.009)	0.067 (0.044 – 0.113)	0.181 (0.129 – 0.298)	0.240 (0.179 0.36)
2020	0.003 (0.002 – 0.005)	0.038 (0.030 – 0.046)	0.787 (0.542 – 1.17)	0.238 (0.166 – 0.356)
2021	0.025 (0.019 – 0.031)	0.093 (0.071 – 0.124)	0.195 (0.139 – 0.262)	0.239 (0.182 – 0.341)
2022	0.031 (0.025 – 0.046)	0.018 (0.013 – 0.024)	0.284 (0.162 – 0.518)	0.187 (0.151 – 0.243)
Ecotope				
Bays	0.014 (0.001 – 0.022)	0.054 (0.030 – 0.123)	0.591 (0.350 – 1.14)	0.311 (0.195 – 0.498)
Central zone	0.013 (0.010 – 0.018)	0.086 (0.068 – 0.110)	0.776 (0.628 – 1.07)	0.355 (0.303 – 0.421)
Near-shore zone	0.019 (0.015 – 0.026)	0.064 (0.051 – 0.083)	0.406 (0.333 – 0.512)	0.240 (0.196 – 0.304)
Rivers	0.017 (0.011 – 0.024)	0.079 (0.055 – 0.111)	0.367 (0.250 – 0.518)	0.326 (0.245 – 0.476)
Central zone – miscellaneous				
Central zone	0.013 (0.009 – 0.019)	0.086 (0.070 – 0.110)	0.776 (0.627 – 1.09)	0.355 (0.299 0.421)
Bays, near-shore zone, rivers mouthes	0.018 (0.015 – 0.023)	0.066 (0.054 – 0.083)	0.422 (0.355 – 0.524)	0.263 (0.223 – 0.309)

Table S2. Assessment of the environmental risk (RQ) for hydrobionts by average concentrations of priority congeners PAEs[1]

PAEs	Hydrobiont	Species	PNEC, μg/L	C average [2], μg/L	RQ
DMP	Algae	Pseudokirchneriella subcapitata	142	0.02	0.0001
	Cladoceran	Daphnia magna	33		0.0005
	Fish	Lepomis macrochirus	50		0.0003
DEP	Algae	Pseudokirchneriella subcapitata	16	0.07	0.0046
	Cladoceran	Daphnia magna	86		0.0008
	Fish	Lepomis macrochirus	16.5		0.0044
D*n*BP	Algae	Pseudokirchneriella subcapitata	142	0.55	0.0039
	Cladoceran	Daphnia magna	33		0.017
	Fish	Danio rerio (Zebra danio)	10		0.055
DEHP	Algae	Pseudokirchneriella subcapitata	0.1	0.30	2.96
	Cladoceran	Daphnia magna	0.77		0.384
	Fish	Lepomis macrochirus	6.0		0.049

Note: [1] – average concentrations of priority PAEs in Lake Baikal waters for monitoring period of 2015-2022; [2] –PNEC – the concentration that is not expected to affect aquatic organisms, data from [**24**,**25**].

Criteria of the assessment of PAEs risk: RQ <0.01 – no risk or a very low level of risk; RQ = 0.01-1.0 – average level of risk; RQ > 1.0 – high level of risk.

Table S3. Assessment of ecological risk (RQ) with increasing concentrations of dominant congeners of PAEs (C, µg/l) in the water of the lake Baikal and at the mouths of the tributaries of the lake

PAEs	Pelagic zone				Under the ice, 5 m		Deep horizons, 1500-1600 m		Selenga shallow water		Tributaries in spring				Near-shore zone			
	St., No 6		St., No 11		St., No 3		St., No 10		St., No 24		St., No 32		St., No 22		St., No 28		St., No 29	
	C	RQ	C	RQ	C	RQ	C	RQ	C	C	C	RQ	C	RQ	C	RQ	C	RQ
D*n*BP	2.5	0.018	1.4	0.010	0.13	0.001	3.7	0.026	11	0,081	0.04	0.000	0.08	0.001	1.3	0.009	8.1	0.057
	2.5	0.077	1.4	0.042	0.13	0.004	3.7	0.112	11	0,348	0.04	0.001	0.08	0.002	1.3	0.039	8.1	0.245
	2.5	0.253	1.4	0.140	0.13	0.013	3.7	0.370	11	1.2	0.04	0.004	0.08	0.008	1.3	0.130	8.1	0.810
DEHP	0.38	3.8	2.2	22	5.8	58	1.2	12	0.70	7.0	0.25	2.5	0.34	3.4	0.24	2.4	0.70	7.0
	0.38	0.494	2.2	2.9	5.8	7.5	1.2	1.6	0.70	0.909	0.25	0.325	0.34	0.442	0.24	0.312	0.07	0.909
	0.38	0.063	2.2	0.367	5.8	0.967	1.2	0.200	0.70	0.117	0.25	0.042	0.34	0.057	0.24	0.040	0.70	0.117

Criteria of the assessment of PAEs risk: RQ <0.01 – no risk or a very low level of risk; RQ = 0.01-1.0 – average level of risk; RQ > 1.0 – high level; RQ >> 1.0 – high level.

Hydrobionts species and quantities values of PNEC (µg/L) are presented in the **Table S2**.
